# Prevalence of pulp stones in patients with cardiovascular disease and type 2 diabetes mellitus: a cross-sectional study

**DOI:** 10.1186/s12903-025-07548-0

**Published:** 2025-12-19

**Authors:** Merve Gökyar, Yeliz Güneş, Hesna Sazak Öveçoğlu

**Affiliations:** 1 Department of Endodontics, Faculty of Dentistry, Marmara University Recep Tayyip Erdoğan Complex Health Campus, 9/3 34854, Başıbüyük/Maltepe/İstanbul, Istanbul, Turkey; 2 Department of Oral & Maxillofacial Radiology, Faculty of Dentistry, Üsküdar University, Istanbul, Turkey

**Keywords:** Cardiovascular diseases, Diabetes mellitus, Endodontics, Pulp calcification

## Abstract

**Background:**

This retrospective cross-sectional study aimed to assess the prevalence of pulp stones in individuals diagnosed with cardiovascular disease (CVD) and/or type 2 diabetes mellitus (DM) using digital panoramic radiographs. Additionally, the study investigated the potential association between these systemic conditions and the presence of pulp calcifications.

**Methods:**

A total of 400 individuals aged 35–70 years were categorized into four equal groups: CVD only, DM only, both CVD and DM, and systemically healthy controls. All archived panoramic radiographs were retrospectively evaluated for the presence of pulp stones. Their distribution was recorded by tooth type, jaw location, and side. Group comparisons were conducted using the chi-square test, with statistical significance set at *p* < 0.05.

**Results:**

The overall prevalence of pulp stones was 59.5% (*n* = 238). The highest prevalence was observed in the healthy control group (69%), followed by the CVD + DM group (65%) and the CVD group (60%), with the lowest prevalence in the DM group (44%). Although differences among groups were statistically significant (*p* = 0.002), these did not indicate a consistent association with systemic conditions. No significant associations were observed regarding anatomical parameters such as jaw (maxilla/mandible), tooth type (first/second molar), or side (right/left).

**Conclusions:**

Although significant intergroup differences were detected, pulp stone prevalence was not elevated in patients with CVD or DM compared with healthy individuals. These findings suggest that pulp stone formation may be multifactorial and not solely determined by systemic conditions. Further research involving larger and more heterogeneous populations is warranted to clarify the potential etiological role of systemic diseases in pulpal calcification.

## Introduction

Pulp stones are calcified structures that develop within the dental pulp. They may be observed in healthy, diseased, or even unerupted teeth [1]. Within the pulp tissue, these calcifications are more commonly found in the coronal region than in the radicular area. Histologically, they can be observed as free-floating, attached to the dentin surface, or embedded within the pulp tissue [2]. Histopathologically, structures that exhibit dentin-like morphology and are surrounded by a layer of odontoblast-like cells are classified as ‘true pulp stones’. In contrast, those containing a central blood vessel and displaying irregular mineral deposits are referred to as ‘false pulp stones’ [3]. The number of pulp stones can range from a single stone to more than twelve within one tooth. These calcified structures range in size from microscopic particles to large masses that may nearly obliterate the pulp chamber [4]. The etiopathogenesis of pulp stone formation is not yet fully understood. However, several factors have been reported in the literature as potential contributors to this process, including advanced age, compromised periodontal health, metabolic disorders such as diabetes mellitus (DM), and particularly cardiovascular diseases (CVD), with atherosclerosis being the most prominent [5–7].

A meta-analytic study conducted by Jannati et al. revealed that pulp stones are present in more than one-third of individuals worldwide [8]. Various radiographic studies have reported that the prevalence of pulp stones varies depending on the study design and the methodology used. For instance, Ranjitkar et al. detected pulp stones in 19.7% of molar teeth in a sample of Australian individuals [9]. Two different studies conducted in Turkey in 2009 reported that the prevalence of pulp chamber calcifications was approximately 5% [10, 11]. In contrast, a study conducted by Kazemizadeh et al. in Iran reported a prevalence rate of 20% [12].

Large pulp stones in the pulp chamber may hinder access to the canal orifices during root canal treatment, thereby complicating the procedure and altering the pulp space anatomy. In particular, stones attached to the dentin surface can deflect the tips of endodontic files or mechanically obstruct their progression along the canal [13].

In recent years, the potential association between pulp stones and systemic diseases has become a noteworthy research topic. This trend offers valuable opportunities for dental professionals to investigate and predict the relationship between pulp stones and various systemic conditions such as DM, nephrolithiasis, hyperlipidemia, autoimmune disorders, and CVD. Pulp stones may be considered potential biomarkers for the detection of systemic diseases. Moreover, the presence of multiple pulp stones detected through routine intraoral radiographs, panoramic imaging, or cone-beam computed tomography (CBCT) may serve as an indicator for systemic disease screening in affected individuals [14]. Some researchers have reported a statistically significant correlation between the presence of pulp stones and the formation of kidney stones [15].

An increased occurrence of pulp stones has been reported in individuals with carotid artery calcifications and in patients diagnosed with DM, compared to healthy individuals [16, 17]. CVD is among the leading causes of morbidity and mortality worldwide. Atherosclerosis is recognized as the primary etiological factor in the development of coronary artery disease, ultimately leading to ischemic heart conditions. Several studies in the literature have suggested a possible association between CVD and the formation of pulp stones [18, 19]. Recent studies have also reported a potential association between hypertension and the formation of pulp stones [20]. Diabetic odontalgia is commonly observed in individuals with type 2 DM. This condition is thought to result from pulpal ischemia and necrosis, which develop due to impaired vascular collateral circulation and weakened immune response. The resulting necrotic environment may serve as a nidus for the initiation of mineralization within the pulp tissue [21].

This retrospective cross-sectional study aimed to evaluate the prevalence of pulp stones in individuals diagnosed with CVD and/or DM using panoramic radiographs. In addition to assessing the potential association between these systemic conditions and pulp stone formation, the study also examines the distribution of pulp stones according to tooth type (first and second molars), jaw location (maxilla/mandible), and side (right/left), as well as the influence of demographic variables such as age and gender. This study was conducted under the hypothesis that individuals diagnosed with CVD and/or DM exhibit a significantly higher prevalence of pulp stones compared to systemically healthy controls.

## Materials and methods

### Ethics approval and consent to participate

The study was conducted in accordance with the ethical standards of the 2008 Declaration of Helsinki and approved by the Ethics Committee for Non-Interventional Research at the Faculty of Health Sciences, Marmara University (Protocol No:81, Date:30.04.2025). The requirement for individual informed consent was waived by an Ethics Committee for Non-Interventional Research at the Faculty of Health Sciences, Marmara University (Protocol No:81, Date:30.04.2025) due to the retrospective nature of the study.

### Study design and participants

Digital panoramic radiographs of individuals aged 35–70 years, archived at the Marmara University Faculty of Dentistry, were used in this study. Radiographs obtained between January and December 2024 were retrospectively reviewed, and a total of 400 individuals who met the predefined inclusion criteria were included in the study.

The study population was divided into four groups, each consisting of 100 individuals:

Group 1: Individuals diagnosed only with CVD,

Group 2: Individuals diagnosed only with DM,

Group 3: Individuals diagnosed with both CVD and DM,

Group 4: Systemically healthy individuals with no history of systemic disease (control group).

The systemic health status of each participant was initially determined from their self-reported medical history archived in patient records. These data were then cross-checked and confirmed using the official electronic health record system provided by the Turkish Ministry of Health. This dual verification ensured the accuracy of diagnoses and the reliability of the group classification.

Only individuals with type 2 DM were included. Type 1 DM cases were excluded to maintain sample consistency, despite the ethics approval being granted for “DM” in general. The CVD category included a range of cardiovascular conditions, such as hypertension, ischemic heart disease, myocardial infarction (heart attack), heart failure, arrhythmias, and valvular heart disease. For the purposes of this study, patients diagnosed with any of these conditions were classified under the CVD group, following verification through patient history and electronic health records.

To ensure balanced group comparisons, a purposive sampling strategy was employed. Eligible individuals were first classified according to their systemic health status (CVD, DM, CVD + DM, or healthy). For each category, 100 participants who met the inclusion criteria and had suitable panoramic radiographs available were consecutively enrolled. The groups were matched for age and gender distribution. Each group was planned to include 50 males and 50 females, and the age distribution was balanced accordingly.

### Sample size and sampling method

The sample size was determined through power analysis based on Cohen’s w coefficient, which is appropriate for categorical data analyzed using the chi-square test. The minimum required sample size was calculated using w = 0.30 (medium effect size), α = 0.05, and power = 0.80, resulting in a total of 88 participants. However, a total of 400 individuals were included in the study to enhance statistical power and improve the reliability of the findings.

Inclusion criteria:

1. Age between 35 and 70 years,
2. Availability of a digital panoramic radiograph archived at the Marmara University Faculty of Dentistry,
3. Sufficient radiographic image quality,
4. Diagnosis of CVD and/or DM, or absence of any systemic disease (for the control group),
5. Eligibility for balanced inclusion across groups in terms of age and gender distribution,
6. Presence of at least 20 natural teeth in the oral cavity (This threshold was applied to ensure that participants had sufficiently preserved dentitions for reliable radiographic assessment and to exclude individuals with extensive tooth loss, who may represent a distinct clinical profile where factors such as advanced periodontal disease, extensive caries, or prior extractions could confound the evaluation of pulp stone prevalence).

Exclusion criteria (Preliminary screening):

1. Individuals with an undefined history of systemic disease or incomplete medical records,
2. Panoramic radiographs with inadequate image quality (e.g., excessive artifacts, image loss, positioning errors),
3. Individuals retrospectively identified as completely edentulous,
4. Individuals with special conditions such as genetic syndromes or generalized calcified pulp disorders.

Exclusion criteria (Post-inclusion):

1. Presence of prior endodontic treatment on the evaluated tooth/teeth (only the affected tooth was excluded from the analysis),
2. Presence of full-coverage crown restorations, post-core structures, large opaque restorations, or metallic components that interfered with radiographic evaluation of the relevant tooth.

### Radiographic evaluation and data collection

All panoramic radiographs were independently evaluated by two experienced observers (MG, YG). The presence and distribution of pulp stones were recorded according to jaw (maxilla/mandible), region (left/right), and tooth type. Possible associations between the presence of pulp stones and systemic conditions—namely CVD and DM—were also investigated. Only the first and second molars were included in the analysis.

All panoramic radiographs were evaluated on a 23-inch Acer monitor (1920 × 1080 pixel resolution) connected to an HP workstation under consistent ambient lighting conditions. Brightness and contrast settings were kept constant throughout the evaluations. Images were viewed using the Romexis software (Planmeca, Helsinki, Finland), which allowed optimal magnification and contrast adjustments when required.

Panoramic radiographs were employed because they are routinely obtained in dental practice, provide a broad overview of both jaws in a single exposure with relatively low radiation, and are suitable for epidemiological and retrospective prevalence studies [6, 16, 19, 20]. The analysis was restricted to first and second molars, as these teeth exhibit the highest prevalence of pulp stones due to their large pulp chambers and complex root canal systems, while also yielding greater diagnostic reliability compared with other tooth groups [7, 9–11]. Participants were not excluded if one or more target molars were missing; in such cases, the available first and/or second molars were evaluated. This approach ensured the inclusion of all eligible individuals while maintaining consistency in the analysis.

### Statistical analysis

All statistical analyses were performed using IBM SPSS Statistics for Windows, Version 27 (IBM Corp., Armonk, NY, USA). Descriptive statistics, including frequencies (n) and percentages (%), were calculated. Differences between the groups were assessed using the Chi-square test, and a significance level of *p* < 0.05 was considered statistically significant. For prevalence estimates, 95% confidence intervals were calculated using the binomial distribution.

To assess inter-observer reliability, 10% of the sample (*n* = 40; 10 patients randomly selected from each group) was re-evaluated independently by two observers, and inter-observer agreement was calculated using Cohen’s kappa (κ = 0.43), indicating moderate agreement.

## Results

**Fig. 1:**
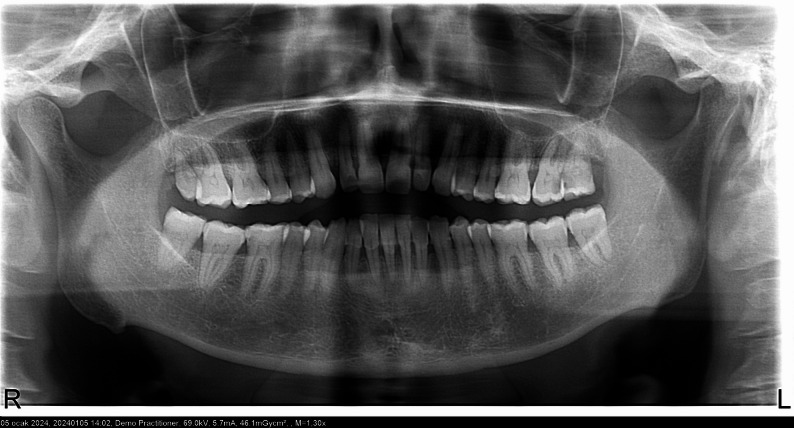
Panoramic radiograph of a patient with diabetes mellitus showing pulp stones in the molar region

**Fig. 2:**
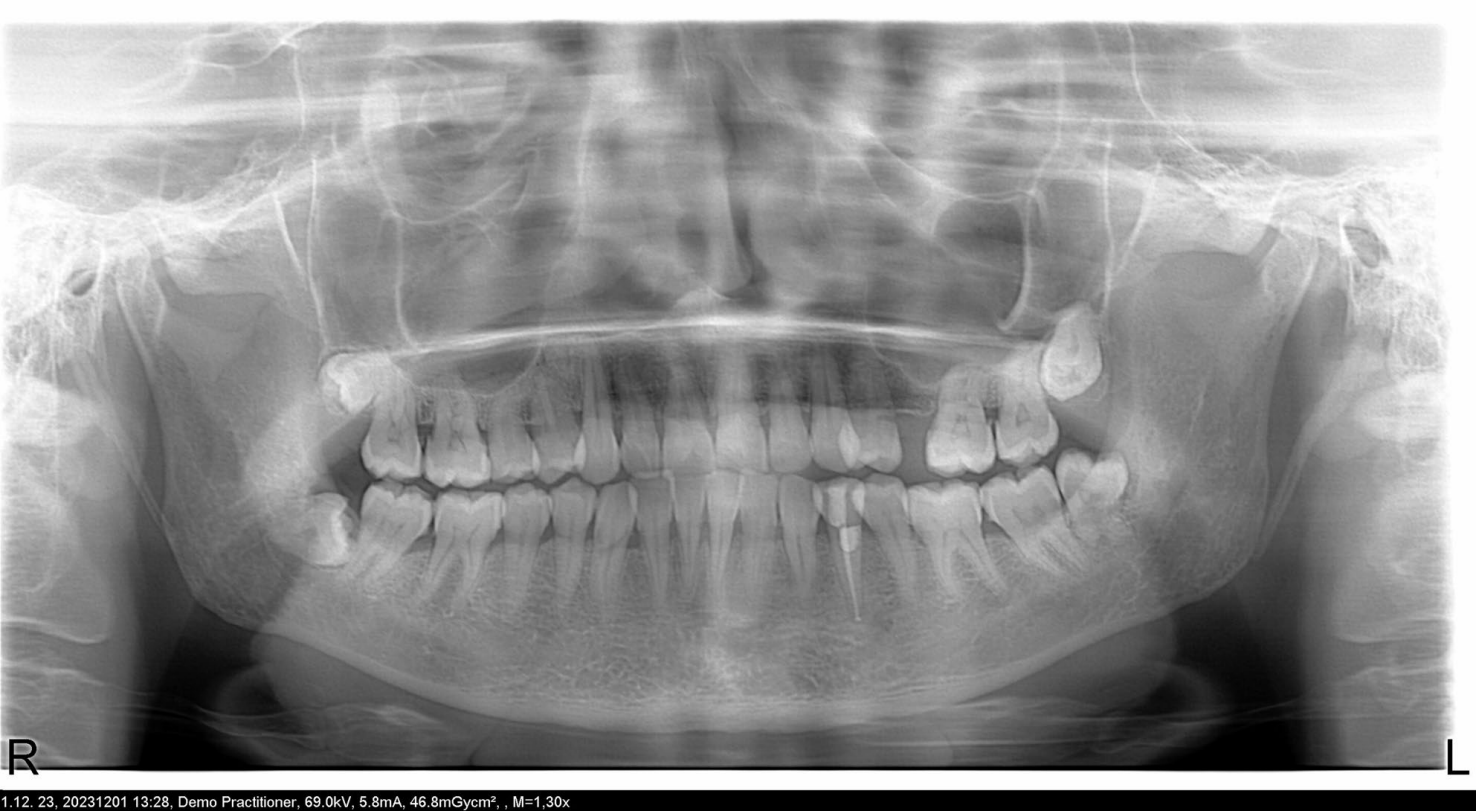
Panoramic radiograph of a systemically healthy individual showing pulp stones in the molar region

In this retrospective study, panoramic radiographs of 400 patients aged between 35 and 70 years were evaluated. The participants were divided into four groups: DM, CVD, DM + CVD, and systemically healthy individuals. Each group comprised 100 patients, with an equal distribution of females (*n* = 50) and males (*n* = 50). The mean age was 44 years in the DM group, 50 years in the CVD group, 46 years in the DM + CVD group, and 50 years in the healthy group. Representative panoramic radiographs demonstrating pulp stones in a diabetic patient and a systemically healthy individual are shown in Figs. 1 and 2, respectively.

The overall prevalence of pulp stones in the study population was 59.5% (95% CI: 54.6–64.2; *n* = 238). Pulp stones were more frequently observed in first molars (59%) compared to second molars (41%). The distribution of demographic and anatomical characteristics according to patient groups is presented in Table 1.

**Table 1:** Patient-level prevalence and tooth-level distribution of pulp stones across study groups (*n* = 400)

		Patient group					
		DM	CVD	DM + CVD	Control	Total	
Mean age, years		44	50	46	50		
Gender	Female	50	50	50	50	200	
	Male	50	50	50	50	200	
Presence of pulp stones	Absent	56	40	35	31	162 (40.5%)	
	Present	44	60	65	69	238 (59.5%)	
Tooth type	1 st molar	79	93	111	138	421 (59.0%)	
	2nd molar	57	57	90	88	292 (41.0%)	
Jaw	Maxilla	88	97	121	135	441 (61.9%)	
	Mandible	48	53	80	91	272 (38.1%)	
Region	Right	66	83	102	119	370 (51.9%)	
	Left	70	67	99	107	343 (48.1%)	

Tooth-level values refer to teeth assessed for pulp stones (*n* = 713)

*DM *diabetes mellitus, *CVD *cardiovascular disease, Control, systemically healthy individuals

A statistically significant association was found between the presence of pulp stones and the patient groups (χ² = 14.98, *p* = 0.002). The highest prevalence was observed in the healthy group (69%, 95% CI: 59.4–77.2), followed by the DM + CVD group (65%, 95% CI: 55.3–73.6) and the CVD group (60%, 95% CI: 50.2–69.1). The DM group showed the lowest prevalence (44%, 95% CI: 34.7–53.8). Comparison of the distribution of pulp stones between first and second molars among the patient groups revealed no statistically significant differences (χ² = 2.187; *p* = 0.535). Similarly, no significant associations were found between patient groups and jaw location (maxilla vs. mandible) (χ² = 1.635; *p* = 0.651) or quadrant (right vs. left side) (χ² = 1.486; *p* = 0.686). These findings are summarized in Table 2.

**Table 2:** Distribution of pulp stones by patient groups

		Patient group						
		DM	CVD	DM + CVD	Control	Total	χ²	p
Presence of pulp stones	Absent	56 (56.0%)	40 (40.0%)	35 (35.0%)	31 (31.0%)	162 (40.5%)	14.981	**0.002**
	Present	44 (44.0%)	60 (60.0%)	65 (65.0%)	69 (69.0%)	238 (59.5%)		
Tooth type	1st molar	79 (58.1%)	93 (62.0%)	111 (55.2%)	138 (61.1%)	421 (59.0%)	2.187	0.535
	2nd molar	57 (41.9%)	57 (38.0%)	90 (44.8%)	88 (38.9%)	292 (41.0%)		
Jaw	Maxilla	88 (64.7%)	97 (64.7%)	121 (60.2%)	135 (59.7%)	441 (61.9%)	1.635	0.651
	Mandible	48 (35.3%)	53 (35.3%)	80 (39.8%)	91 (40.3%)	272 (38.1%)		
Region	Right	66 (48.5%)	83 (55.3%)	102 (50.7%)	119 (52.7%)	370 (51.9%)	1.486	0.686
	Left	70 (51.5%)	67 (44.7%)	99 (49.3%)	107 (47.3%)	343 (48.1%)		

Values are presented as n (%). Tooth-level values refer to teeth assessed for pulp stones (*n* = 713)

*DM *diabetes mellitus, *CVD *cardiovascular disease, Control, systemically healthy individuals

## Discussion

In the present study, the prevalence of pulp stones was evaluated using panoramic radiographs in individuals with CVD and/or DM, and their potential association with pulp stone formation was investigated. Pulp stones are typically detected incidentally on radiographic examinations as calcified structures [22]. In recent years, with growing scientific interest in the etiopathogenesis of pulp stones, several studies have reported significant associations between pulp stones and certain systemic diseases [3, 23].

Studies using two-dimensional radiographic techniques such as intraoral periapical (IOPA), bitewing, and orthopantomographic (OPG) radiographs have reported a wide prevalence range for pulp stones, from 8% to 90% [8]. In the present study, the prevalence was 59.5%. Variations across studies may result from differences in sample size, diagnostic methods, demographic factors, ethnicity, and geographic region [24]. Histological examination is widely regarded as the gold standard for detecting pulp calcifications because it enables the identification of small or early mineral deposits that may not be visible on radiographic imaging [25]. However, its invasive nature restricts its use to extracted teeth or ex vivo research designs, limiting its applicability in large-scale epidemiological studies. Although CBCT provides superior three-dimensional visualization and improved diagnostic accuracy compared with two-dimensional radiographs, very small calcifications may still remain undetectable, leading to lower sensitivity relative to histological analysis [17]. These methodological considerations explain the preference for CBCT in clinical and epidemiological studies despite its higher cost and radiation dose.

A recent systematic review evaluating 46 studies reported that 78.3% of the included studies found a positive association between pulp stones and systemic diseases [26]. However, in the present study, the highest prevalence of pulp stones was observed in healthy individuals. Although this finding appears to contradict the literature, it may be explained by several factors, including study design, exclusion criteria (e.g., the omission of carious or restored teeth), age range, and population-specific characteristics. In addition, pulp stone formation is multifactorial; even in systemically healthy individuals, genetic predisposition, occlusal loading, and subclinical pulpal inflammation may contribute to calcification [2, 4, 10]. The exclusion of teeth with full-coverage crowns, post-core structures, or radiopaque restorations that interfered with radiographic evaluation might also have led to an underestimation of pulp stone prevalence across all groups, since such teeth could not be reliably assessed. Furthermore, population-specific factors such as oral health behaviors, dental care accessibility, and the relatively balanced age distribution in our sample could also explain why pulp stones were more prevalent among healthy individuals. Taken together, these factors may partly explain the unexpectedly high prevalence of pulp stones in the healthy control group. Therefore, pulp stones should not be regarded solely as markers of systemic disease but rather as outcomes of a multifactorial process influenced by systemic, local, biological, and environmental factors. For example, local factors such as caries, restorations, and periodontal disease [10], along with biological determinants including aging [2] and systemic metabolic changes [21], may also contribute to their development.

Nowadays, pulp stones are considered potential risk indicators for the early detection of CVD [19]. Calcifications observed in the kidneys, joints, dental tissues, and atherosclerotic plaques are largely composed of calcium phosphate crystals, which may trigger immune responses in vascular walls and contribute to ischemic heart disease pathogenesis [18]. Srivastava et al. reported that individuals with CVD had nearly threefold higher odds of pulp stones compared with healthy individuals [17]. Nayak et al. also observed the highest prevalence in CVD patients, with increased frequency in those with DM and autoimmune disorders [7]. Edds and Khojastepour detected pulp stones in 74% and 68.2% of ischemic heart disease patients, respectively [6, 14], while Ezoddini-Ardakani et al. reported a prevalence of 67.3% [19]. Histopathological data by Bernick et al. further supported vascular narrowing in CVD patients [27]. In contrast, the present study found pulp stones in 60% of the CVD group and 65% of the CVD + DM group, but the highest prevalence occurred in the healthy controls (69%). These results suggest that pulp stone formation may be influenced not only by systemic diseases but also by factors such as age, genetic predisposition, environmental influences, and radiographic methods. Differences in disease duration, systemic control, and diagnostic criteria may also explain discrepancies across studies [8, 17]. Overall, our findings highlight the multifactorial nature of pulp stone formation and underscore the need for studies with larger, more diverse populations. Supporting this, Yilmaz et al. found no significant association between hypertension and pulp stones [28], and Gulsahi et al. similarly failed to identify significant systemic correlations, though disease groups were not clearly specified [11].

The dysfunction caused by DM in the immune system negatively affects both oral and systemic health, leading to a significant decline in overall quality of life [29]. In a CBCT-based study, Srivastava et al. reported that individuals with DM had a 1.81-fold higher risk of developing pulp stones compared to healthy individuals [17], attributing this to DM-induced obliterative endarteritis and aging-like pulpal changes. However, the present study found a significantly higher prevalence of pulp stones in healthy individuals than in those with DM, which does not align with these results. These contradictory findings may stem from methodological differences and heterogeneity across studies. Variables such as age distribution, disease duration, metabolic control, and imaging techniques (e.g., CBCT, periapical radiographs) can directly influence prevalence estimates. Indeed, Srivastava et al. reported 9.01% in diabetic patients [17], while Nayak et al. found 7.69% [7]. In contrast, the prevalence in the diabetic group was 44% in the present study. Such discrepancies may also reflect differences in diagnostic criteria, inter-examiner variability, and sample sizes across studies.

The case-control study Romano et al. was consistent with the present findings [30], showing no significant association between pulp stones and CVD or DM but a higher prevalence in individuals with kidney stone history. In contrast, Tarım Ertaş et al. found no correlation between renal calculi and pulp stones [31]. Likewise, Horsley et al. reported that pulpal calcifications are not reliable predictors of carotid artery calcification [32].

In the present study, individuals aged 35–70 years were included. This interval was chosen because pulp stones are rare under 35, while in those over 70, extensive tooth loss often limits radiographic assessment [11]. Moreover, this age range captures both higher systemic disease prevalence and adequate tooth retention, making it suitable for investigation. Age averages were kept comparable across groups to minimize confounding. Age-related biological changes—such as reduced cellularity, increased mucopolysaccharide concentration, and fibrous tissue accumulation—may also promote pulpal calcification [33].

This study has some limitations that should be considered. First, the use of panoramic radiographs may underestimate the true prevalence of pulp stones, as very small calcifications cannot be detected without histological confirmation. While CBCT offers greater diagnostic accuracy, its higher cost and radiation dose limit its application in large-scale epidemiological studies. Second, the analysis was restricted to first and second molars, which may limit the generalizability of the findings to the entire dentition. Third, no information was available on the duration or severity of systemic diseases (e.g., long-standing vs. newly diagnosed DM or CVD), which may have influenced the prevalence of pulp stones. Fourth, the retrospective single-center design and inclusion of a Turkish population only may restrict the external validity of the results. Additionally, the study employed a purposive sampling strategy with equal group sizes (*n* = 100 each) to enable balanced statistical comparisons. While this approach enhanced internal validity, it may not reflect the natural prevalence of systemic conditions in the broader population. Furthermore, the groups were deliberately matched for age and gender (50 males and 50 females per group) to ensure balanced comparisons. While this strategy strengthened internal validity, it may limit real-world applicability, as strict matching does not reflect the natural distribution of systemic conditions in the general population. Nevertheless, the relatively large and well-balanced sample provides meaningful insights into the prevalence and distribution of pulp stones in patients with CVD and DM. Finally, intra-observer reliability was not assessed in this study, which represents a methodological limitation that may have affected the repeatability of the radiographic measurements. Furthermore, the inter-observer reliability demonstrated only moderate agreement (κ = 0.43), which may have influenced the precision and reproducibility of the study findings and should be taken into account when interpreting the results.

## Conclusion

Within the limitations of this retrospective study, pulp stones were frequently observed in both systemically healthy individuals and patients with CVD or type 2 DM, with the highest prevalence detected in the control group. These findings suggest that pulp stone formation is a multifactorial process and may not represent a reliable indicator of systemic disease. Future multicenter investigations with larger and more diverse populations, incorporating advanced imaging modalities, are warranted to clarify the potential association between systemic conditions and pulpal calcifications.

## Data Availability

The datasets used in the current study are available from the corresponding author upon reasonable request.
